# THE IMPORTANCE OF THE LONG-TERM CARE WORKFORCE ON QUALITY OUTCOMES FOR INDIVIDUALS RECEIVING SERVICES

**DOI:** 10.1093/geroni/igz038.1654

**Published:** 2019-11-08

**Authors:** Lindsay Schwartz, Howard Degenholtz, Amy M York

**Affiliations:** 1 American Health Care Association/National Center for Assisted Living, Washington, District of Columbia, United States; 2 University of Pittsburgh, Pittsburgh, Pennsylvania, United States; 3 Eldercare Workforce Alliance, Washington, District of Columbia, United States

## Abstract

A strong, supported long-term care (LTC) workforce is vital to quality outcomes of individuals receiving LTC. With the sector facing issues around recruitment and retention, it is important to understand factors impacting the workforce. This symposium includes four presentations, a mix of both quantitative and qualitative research. First, Scales and colleagues will provide an overview of the workforce crisis using extensive policy analysis of home and community-based services (HCBS) in the US. Factors impacting the HCBS direct care workforce (DCW), including training, supply and demand, models of care and compensation, will be addressed. Next, Carder et al. will compare workforce recommendations from the 2003 Assisted Living Workgroup (ALW) report to current regulations. Many states have incorporated recommendations including criminal background checks and training while few have required staff performance evaluations and policies to improve retention. Morgan et al. examine AL residents’ care convoys’ impact on resident outcomes utilizing data from interviews with AL staff, external health care professionals, residents and family members (n=219). Policies, practices, work overload, time constraints, lack of training and turnover impacted DCW involvement in care convoys. Bender et al. analyze data from 14 DCWs and 16 executive directors from 4 ALs to examine how staff implement and understands end-of-life care policies and procedures. Limited training and communication around death present opportunities for improvement to support DCWs experiencing grief and bereavement. The discussant will address the importance of workforce as part of the network providing quality of care and improving quality of life of individuals receiving LTC.

